# Immigration detention of children: a systematic review and meta-analysis of physical and mental health impacts

**DOI:** 10.1007/s00787-025-02832-4

**Published:** 2025-08-27

**Authors:** Bafreen Sherif, Debbie C. Hocking, Mohammad Asghari-Jafarabadi, Susan Rees, Letizia M. Affaticati, Suresh Sundram

**Affiliations:** 1 Department of Psychiatry, School of Clinical Sciences, Faculty of Medicine, Nursing and Health Sciences, Monash University, Clayton, VIC Australia; 2 Cabrini Asylum Seeker and Refugee Health Hub, Cabrini Outreach, Northcote, VIC Australia; 3 Cabrini Research, Cabrini Health, Malvern, VIC Australia; 4 School of Public Health and Preventive Medicine, Monash University, Melbourne, VIC Australia; 5 Discipline of Psychiatry and Mental Health, School of Clinical Medicine, Faculty of Medicine and Health, UNSW, Sydney, NSW Australia; 6 Department of Medicine and Surgery, University of Milan Bicocca, via Cadore 38, Monza, 20900 Italy; 7 Mental Health Program, Monash Health, Clayton, VIC Australia

**Keywords:** Refugees, Asylum seekers, Immigration detention, Physical health, Mental health

## Abstract

Upper-middle and high income countries employ immigration detention for asylum-seeking/refugee (ASR) children despite documented health risks. We sought to assess the physical and mental health impacts of detention on ASR children in these settings. A systematic review of 15 studies (*N* = 4,890 children) from 2,512 screened records was undertaken. Relevant databases were searched from 1808 to October 2024 for papers using the search terms ‘unaccompanied/separated/children/minors/adolescents,’ and ‘war/trauma/asylum/refugee,’ and ‘mental illness/ PTSD/depression/ distress, depressive symptoms/ anxiety/physical health/ health/risk factors,’ as well as ‘detention/detain/imprison/incarcerate’. Joanna Briggs Institute tools assessed quality. Strengths and Difficulties Questionnaire (SDQ) data from three studies (*n* = 239) underwent random-effects meta-analysis as the primary measure of social-emotional and behavioural issues. Duration of detention ranged from 2 weeks to 36 months. Prevalence of PTSD ranged from 6.5% (brief detention < 30 days) to 100% (prolonged detention ≥ 12 months). MDD was reported in 44–95% of children, and suicidal ideation/self-harm in 25–57%. Developmental delays were observed in 75% of children detained ≥ 12 months. Anxiety disorders (45–50%) included separation anxiety and generalised anxiety. The mixed model meta-analysis revealed severe mental health burdens with detained children exhibiting total difficulties scores twice the normative population mean (16.63 vs. 7.1–8.4, 95% CI: 11.19–22.07) with elevated subscale scores for emotional symptoms (5.40 vs. 1.6–2.1, 95% CI: 3.79–7.01), conduct problems (2.93 vs. 1.3–1.6, 95% CI: 1.53–4.32), and peer problems (3.34 vs. 1.4–1.6, 95% CI: 2.17–4.51) (I² >75% for all mental health outcomes). Physical health impacts included malnutrition (24–56%), dental caries (21–54%), vitamin D deficiency (51%), and somatic complaints (e.g., headaches, enuresis). Systemic risk factors—detention duration, family separation, and cumulative trauma—exacerbated harm, with parental mental illness (54%) and dehumanising conditions (e.g., numeric identification) amplifying risks. Comparative data highlight resettled children’s recovery trajectories when provided stability and community support. Most studies were cross-sectional, limiting the ability to assess causality. This review consolidates evidence that establishes a connection between immigration detention and the manifestation of mental disorders, categorising it as an instance of early-life adversity. All fifteen studies identified detrimental consequences, and a meta-analysis of three studies revealed significant emotional distress and behavioural issues. These findings underscore the urgent need for policy reforms to abolish prolonged detention, prioritise family unity, and implement trauma-informed care to mitigate irreversible health consequences for ASR children. Policymakers must recognise these impacts and refrain from detaining and separating children and families.

**Systematic review registration:** https://www.crd.york.ac.uk/prospero/, registration number CRD42022328867.

**Supplementary Information:**

The online version contains supplementary material available at 10.1007/s00787-025-02832-4.

## Introduction

The past decade has witnessed an unprecedented escalation in global forced displacement, driven by armed conflict, persecution, and systemic human rights violations [1]. Children under the age of 18 comprise approximately 40% of forcibly displaced populations, equating to around 47 million minors [1]. Unique vulnerabilities distinguish children from adults, rooted in physical, cognitive, and emotional development. This developmental immaturity renders them acutely sensitive to psychological trauma and environmental instability. Children and adolescents exposed to trauma are vulnerable to age-specific developmental risks, including impaired attachment formation in infancy, problems with identity consolidation during adolescence, and reduced neuroplasticity throughout childhood [2, 3].

ASR children additionally face compounded vulnerabilities, including mental health difficulties [4–6], enduring traumatic pre-migration exposures, perilous migratory journeys and post-migration stressors such as family separation, discrimination and restrictive immigration policies [7]. Children and adolescents who are refugees exhibit a greater prevalence of mental disorders in comparison to their non-refugee counterparts. The impaired psychological well-being among refugee children and adolescents can predominantly be ascribed to experiences of warfare, transitional stresses such as detention, and challenges associated with acculturation [8]. Barghadouch et al. [9] posit that experiencing childhood in a refugee context may lead to an increased, long-term prevalence of psychotic and neurotic disorders in early adulthood. A cohort of 15,264 young adult refugees who obtained residency permits was matched at a ratio of 1:6 based on age and sex with 99,313 Danish-born children. The refugees exhibited higher relative risks (RRs) of psychotic disorders (RR: 1.81, 95% CI: 1.41–2.32) and nervous disorders (RR: 1.28, 95% CI: 1.14–1.43) compared to their Danish counterparts. Moreover, being over 12 years of age at the time of obtaining residency in Denmark was identified as a predictor of increased psychiatric morbidity within the study population, which is interpreted as an indicator of greater cumulative adversity, suggesting that older refugees have been exposed to a higher number of risk factors for psychiatric disorders. The protective effect associated with being younger during the initial years of resettlement may be related to an enhanced capacity to adapt to the host country [10].

Another systematic review of 15 studies examining post-traumatic stress disorders, depression, and anxiety in unaccompanied refugee minors (URMs) exposed to war-related trauma highlighted the high prevalence of post-traumatic stress disorders (PTSD), depression, and anxiety among URMs [11]. Separation from family members, death of parents and close relatives, level of exposure to armed conflicts, and threats to a person were the most frequent stressful life events (SLE) among URMs before migration. The total number of SLE experienced was noted to be a risk factor for PTSD, depressive symptoms, and anxiety and the severity of traumatic events was associated with the severity of psychological outcomes. For example, the death of a relative and post-migration stress showed more severe PTSD and depression outcomes among URMs. In addition to being separated from loved ones and witnessing armed conflicts and wars before migration, URMs worried about their family and their future in the host country. Twenty-eight percent of URMs who were not diagnosed with PTSD at the first assessment were diagnosed with PTSD after two years of resettlement [11].

Exposure to pre-migration violence, female gender (as a factor associated with internalising or emotional difficulties), unaccompanied status, experiences of perceived discrimination, exposure to post-migration violence, multiple relocations within the host country, parental exposure to violence, insufficient financial support, residing in a single-parent household, and the presence of parental psychiatric disorders have all been identified as significant risk factors in a systematic review comprising 44 studies involving 5776 displaced children and adolescents that affect mental health outcomes in forcibly displaced minors [10].

ASR children may also exhibit significant and varied physical health burdens, with a systematic review encompassing 53 population-based studies revealing significantly elevated estimated prevalence rates for anaemia (14%), hemoglobinopathies (4%), chronic hepatitis B (3%), latent tuberculosis infection (11%), and vitamin D deficiency (45%) upon their arrival in host countries. Additionally, approximately one-third of refugee children presented with intestinal infections and nutritional issues, ranging from wasting and stunting to obesity [12]. Moreover, restricted access to healthcare in host countries complicates the management of pre-existing health conditions [13].

At the same time, adverse childhood experiences (ACEs) are associated with poor outcomes in a range of physical health, mental health, and social parameters in adulthood [14]. The factors contributing to adverse health outcomes in child ASR populations, including exposure to violence, interruptions in educational opportunities, family separations, and detrimental familial dynamics, have been thoroughly documented in existing literature [15–20]. Notably, post-migration stressors such as detention, discrimination, and parental mental illness increasingly predict psychological harm over time, particularly when pre- and peri-migration traumas (e.g., war exposure and family separation) are no longer ongoing. This temporal association highlights the cumulative impact of systemic adversities in host countries, which frequently surpasses the influence of prior traumatic events [10, 21–27].

Many host countries are becoming increasingly reluctant to provide protection to those seeking asylum, adopting restrictive immigration practices driven by deterrent policies despite a growing body of evidence that these policies harm ASR, particularly for children [28–32]. From a rights-based perspective, the immigration detention of children constitutes a clear violation of international legal norms. The Convention on the Rights of the Child (CRC), ratified by 196 countries, stipulates under Article 37 that children should only be detained as a measure of last resort and for the shortest appropriate duration [33, 34]. Article 3 mandates that the best interests of the child be a primary consideration in all actions concerning them, and Article 22 guarantees specific protections for refugee and asylum-seeking children [33, 34]. These principles are reinforced by the International Covenant on Civil and Political Rights (ICCPR), which prohibits arbitrary detention (Article 9) [35] and is supported by UN Committee General Comments that unequivocally condemn the immigration detention of children [36, 37]. The UNHCR has consistently advocated for community-based alternatives to detention and affirms that children should never be detained on the basis of their own or their parents’ migratory status [38].

Despite the frameworks that establish both a legal and moral obligation to cease the detention of children for immigration purposes, and which situate this issue within a broader political and societal framework of child protection, these statements accurately reflect the *de jure* position of international human rights law. However, actual practice (de facto compliance) remains inconsistent across jurisdictions. The detention of ASR children is a practice observed globally, encompassing high-, middle-, and low-income categories, thereby establishing it as an issue of significant global concern [28, 39]. It is posited that the vulnerability of children in immigration detention to the development of mental health disorders arises from a complex interplay of factors associated with pre-, peri-, and post-migration experiences [19, 40–42].

Post-migration detention has been noted as particularly detrimental to the mental health of children, with the prompt yet judicious resolution of asylum claims suggested as a means to mitigate the duration of uncertainty, insecurity, and the associated distress experienced by these children. Insecure asylum status is correlated with a range of psychological issues. Experiences during immigration interviews and subsequent detention can be profoundly distressing for children, exacerbating prior adverse experiences with authority and placing them in situations that may be perceived as more detrimental than the challenges encountered prior to migration [10].

The existing body of literature has consistently documented elevated levels of psychological distress among detained ASR children [29, 40, 43–47]. This distress is accompanied by well-documented psychopathological conditions, including major depressive disorder (MDD) with a prevalence ranging from 20% to 50%, anxiety disorders with a prevalence ranging from 20% to 40%, and PTSD with a prevalence ranging from 10% to 50% [7, 48–51]. Prevalence estimates of any emotional disturbance vary between 33.3% and 80% [28, 29, 32, 40, 47, 52, 53]. There is a dose-response relationship between detention duration and negative mental health outcomes among child ASR [30, 31, 52–58]; however, even brief periods of detention have been shown to have deleterious effects [40, 45].

The effects of immigration detention on the physical health of child ASR are not extensively documented, and the prevalence rates exhibit considerable variability. Nevertheless, 89%–100% of child ASR appear to encounter physical health challenges, with malnutrition affecting between 24% and 60% and dental disease impacting between 21% and 59% [59–61]. Furthermore, developmental concerns are raised by 23%–60% of parents [32, 52]. Additionally, children in detention exhibit greater health needs than their counterparts in the host nation, with skin, respiratory, and urological issues among the primary complaints necessitating healthcare visits [62].

While previous systematic reviews have confirmed high rates of mental disorders among ASR children [63–65], the physical and psychological health of ASR children exposed to immigration detention has yet to be systematically reviewed. The purpose of this systematic review and meta-analysis is to provide a contemporary synthesis of the evidence relating to the effects of immigration detention on the physical and mental health of child ASR, specifically aiming to document the prevalence and types of physical and mental health conditions among child ASR who have experienced immigration detention, as well as to identify associated factors.

This review distinguishes between child asylum seekers (minors undergoing refugee status determination under Article 1(A)(2) of the 1951 Refugee Convention) and recognised refugee children (those granted formal protection under Article 1(C)) [66]. While the latter is unequivocally protected from detention under Article 37 of the Convention on the Rights of the Child (CRC) [34], asylum-seeking children face permissible—though internationally discouraged—detention, with at least 77 countries having laws and policies that allow them to be detained based on their legal or migratory status [67]. Every year, a minimum of 330,000 children worldwide are deprived of their liberty based on their legal or migratory status or that of their parents. The lack of precise data indicates a probable significant underestimation. Despite the commitments made by numerous countries to eliminate child immigration detention, the reality is that even in certain nations where legislation does not support immigration detention, such practices continue [67].

In 2022, the United Nations Task Force on Children Deprived of Liberty urged States to undertake efforts to gather disaggregated and harmonised data pertaining to the immigration detention of children. Additionally, it emphasised the necessity for child rights-based alternative solutions, with the stipulation that such data be made publicly accessible and disaggregated by age, sex, country of origin, citizenship, disability status, and the accompanying status of the child [67].

## Methods

We conducted a systematic review and meta-analysis following the modified version of the PRISMA guidelines [68]. The protocol was registered in the International Prospective Register of Systematic Reviews (PROSPERO), registration number CRD42022328867.

### Search strategy and study selection

The search was conducted in December 2021 and repeated in October 2024 by three members of the research team (BS, DH and LMA). Relevant studies were identified through electronic searches of Embase (1980 to 2024 week 43), OVID MEDLINE (1946 to 2024 week 43), APA PsychINFO (1806 to 2024 week 43), CINAHL Plus (1937 to 2024 week 43), Global Health (1910 to 2024 week 43), APA PsycARTICLES (1967 to 2024 week 43), Social policy and practice (from 1981 to 2024 week 43), Cochrane Library (from 1993 to 2024 week 43), Scopus, Open Grey and advanced Google search of.org websites until ten consecutive pages did not include relevant pages (last searched October 2024). The search string followed that of Von Werthern et al. (2018) [69] (supplementary material). Searches were limited to English language only. No other restrictions were applied to the searches.

Electronic searches were supplemented with the screening of reference lists of included primary studies.

By applying Boolean operators described in the Cochrane guidelines (2024 [70]), different combinations of terms were employed for the search in the databases:

1. unaccompanied or separated or children or minors or adolescents and.
2. war or trauma or asylum or refugee and.
3. mental illness or post-traumatic stress disorder (PTSD) or depression or distress or depressive symptoms or anxiety or physical health or health or risk factors and.
4. Detention or detain or imprisoned or incarcerate or temporary protection or custody or prison or jail.

### Selection criteria

The UN Convention on the Rights of the Child definition of a child (below the age of 18 years) was applied across the sample [33].

Quantitative studies were included if they: (i) sampled children detained for migration-related reasons (e.g. unauthorised entry, visa overstays) at the time of the study or after release; (ii) used randomised control or quasi-experimental design with matched samples to minimise selection bias; (iii) assessed health outcomes linked to detention; (iv) provided quantitative results from published peer-reviewed research in English; (v) involved children aged below 18 years.

Studies were excluded if they: (i) did not analyse the health impact of immigration detention; (ii) Focused on detention for non-migration-related unlawful acts (e.g. criminal offences), excluding migration-specific grounds such as unauthorised entry or visa overstays; (iii) exclusively included participants not deprived of freedom of movement, (e.g. community dwellings, reception centres); (iv) participant age criteria were not met (participants ≥ 18 years of age); (v) were meta-analyses, editorials, or scoping reviews. No restrictions were placed on the origin of detainees, country of publication, or method of assessing health outcomes.

### Data extraction and synthesis

Two authors (BS, DH) screened the titles and abstracts of identified articles against the inclusion criteria. The full text of each study was reviewed, and in cases where it was difficult to determine the study’s relevance to the systematic review, a third author (SS) was consulted. Three authors (BS, DH, SS) screened the full texts, resolving disagreements through discussion. Extracted data from included studies were compiled into a standardised Microsoft Excel spreadsheet by two authors (BS, DH), overseen by a third (SS). For each study, the first author, publication year, study design, measures of physical and mental health, and length of immigration detention were extracted. Prevalence rates of disorders, not limited to PTSD, depression, and anxiety, but also covering neuropsychiatric illnesses and relevant physical health data were collected. Additional data included refugee/asylum seeker status, country of origin and host country, length of detention, traumatic events experienced, mean age, and male-to-female ratio where available.

### Quality assessment of included studies

A quality assessment was conducted for all 15 studies using the Joanna Briggs Institute Checklists (JBI) [71, 118]. Three review authors (BS, DH, LMA) conducted an independent, blinded assessment for each included study. For the cross-sectional studies included in the review (*n* = 10), the following domains were evaluated: Inclusion Criteria, Subjects and Setting, Exposure, Condition, Identification of Confounders, Strategies for Confounders, Outcomes, and Statistics. Additionally, the following criteria were employed to assess the quality of the prevalence studies included in the review (*n* = 5): Sample Frame, Sampling and Recruitment, Sample Size, Description of Subjects and Setting, Data Analysis conducted with adequate coverage, Valid Methods for Identification of Condition, and Standard/Reliable Measures of Condition. Disagreements were resolved by consensus. A detailed analysis of quality appraisal based on the JBI checklist is provided in the supplementary text (Table 2).

### Statistical analyses

Analyses were performed using STATA18 (StataCorp, College Station, Texas, USA). Study reporting followed PRISMA guidelines [72]. Mean and standard deviation of primary outcomes were extracted, and random-effects meta-analyses were conducted with a restricted maximum likelihood approach [73]. A random-effects model was chosen to account for potential unregistered or unpublished studies. Between-study heterogeneity was assessed with the Cochran Q test, Tau squared, and I-squared statistics. Publication bias was evaluated using Egger’s and Begg’s tests, while individual study effects on the overall effect size were examined through a leave-one-out method.

### Meta-analysis outcome Measure – The strengths and difficulties questionnaire (SDQ)

The Strengths and Difficulties Questionnaire (SDQ) is a brief, 25-item measure of behavioural and emotional difficulties [74, 75] that can be used to assess mental health problems in children and young people aged 4–17 years and was cross-compared across detained ASR children. The 25 items generate five symptom subscales (scored 0–10) —conduct problems, hyperactivity-inattention, emotional symptoms, peer problems, and prosocial behaviour [74]. All subscales except for prosocial behaviour are summed to produce a total difficulties score, ranging from 0 to 40. With the exception of the (reverse-scored) pro-social subscale, higher scores correspond to increasing difficulties. Each score can be compared against population norms and categorised as “normal”, “borderline”, and “abnormal” [74–77]. Cut-off scores were originally designed such that roughly 80% of children’s scores fall within the “normal” range, 10% within the “borderline” range, and 10% within the “abnormal” range [74]. The SDQ has demonstrated high sensitivity and specificity in assessing the social-emotional well-being of migrant and refugee children [75]. In both epidemiological and clinical studies, high SDQ scores are routinely employed to screen for increased risk of mental illness [75, 78].

## Results

### Study selection

**Fig. 1:**
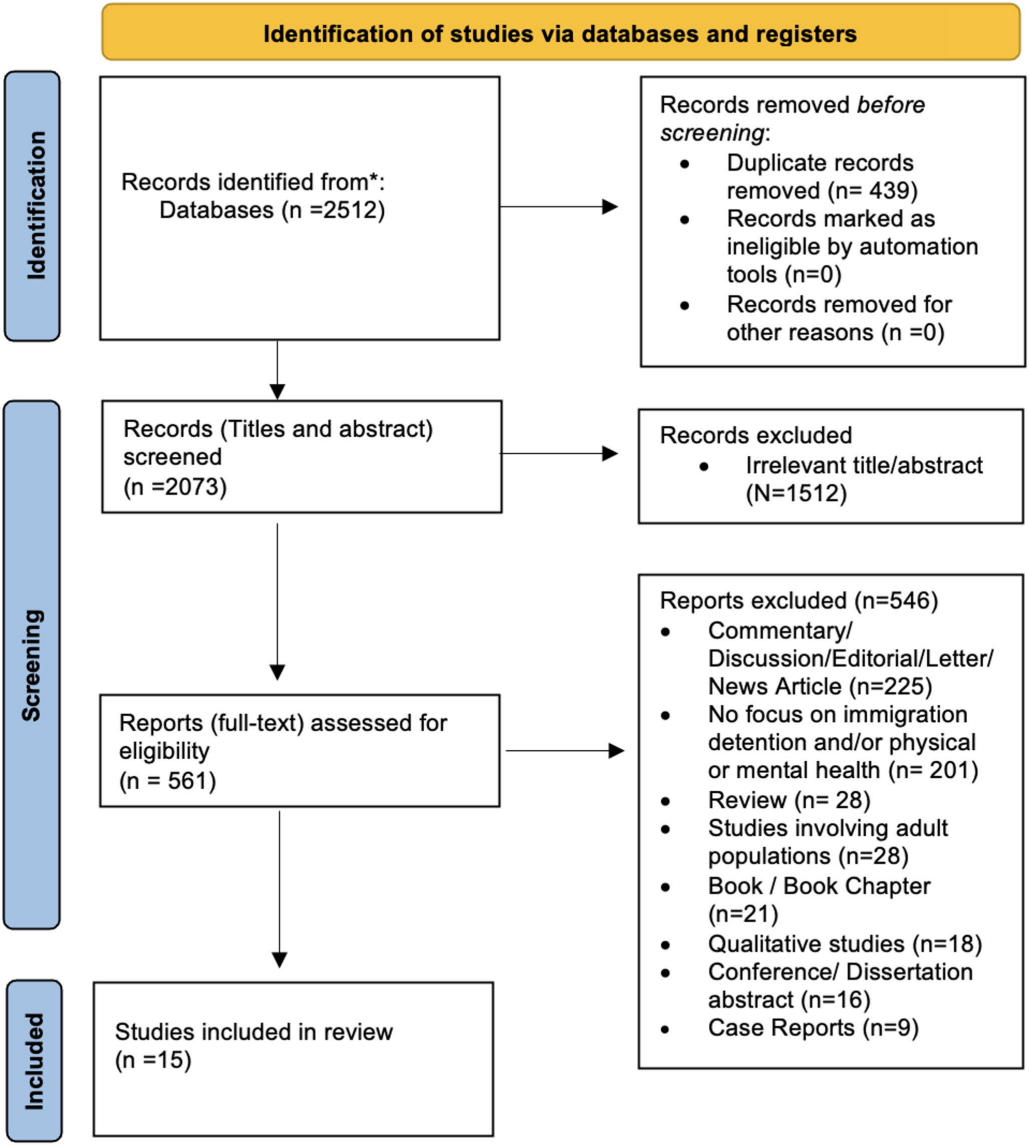
PRISMA flow diagram for systematic review

The entire search, including electronic databases and other sources, yielded a total of 2,512 articles. A PRISMA-style flowchart outlining the search results and selection criteria of studies is provided in Fig. 1. After the removal of 439 duplicates and 1,512 titles and abstracts screened as irrelevant, a further 561 articles were screened through full-text retrieval for eligibility. Fifteen studies pertaining to child and adolescent populations met the inclusion criteria.

### General characteristics of the included studies

Table 1 presents the study design, sample and participant characteristics, country of origin, outcome(s) of interest, method of assessing the outcome(s), and a description of the main results from these 15 studies. The 15 eligible studies provided data on 4,890 child ASR, with sample sizes ranging from 20 to 277.

ASR samples were drawn from Australia (*n* = 8) [32, 43, 59–62, 76, 79], Libya (*n* = 1) [80], the United Kingdom (*n* = 2) [6, 40], and the United States of America (*n* = 4) [19, 81–83]. Study size, participants, outcomes and study design, along with the 21 different diagnostic measures used to assess health outcomes, are noted in Table 1.

The ages of included children ranged from 0 months to 22 years, as one study included 48 infants (25 infants had been born into detention, and 20 women were pregnant) [32] and another [60] sampled participants who were ≤ 18 years at detention entry but aged during prolonged detention (52% held on Nauru ≥ 4 years). Two studies did not include the ages of the sampled children [62, 80].

Six studies included unaccompanied minors (*N* = 170); [80] (*n* = 99) [6], (*n* = 35) [81], (*n* = 19) [32], (*n* = 15) [43], (*n* = 1), and [60] (*n* = 1) of which two studies exclusively examined currently ([80]) or previously detained [6] unaccompanied minors.

During detention, instances of family separation were recorded in two studies [60, 61], wherein 11 and 58 children, respectively, were separated from ≥ 1 parent while in immigration detention. Single-parent households were reported in [40] (83.3% of the sample; 75% single mothers) [43], (10% of the sample) [84], (36.6% of the sample) [83], and 11 and 18 adolescents had left their mothers and fathers, respectively.

In one study [82], all 84 children who had been separated from their parents at the United States-Mexico border were reunited with their families by the time the interviews were conducted, which occurred two days after their release from an average detention period of seven days.

**Table 1:** Characteristics and key findings of the impacts of immigration detention on the health of child asylum seekers/refugees (*N* = 15)

Author and year	Study Design	Sample type	Age	Gender	Detention duration	Country of origin	Country of study	Outcome of interest	Method of assessing outcome	Assessment of outcome in relation to time since detention	Mental Health Conditions	Physical Health Conditions
Amarasena, 2023 [60]	Analytical cross-sectional study	62 asylum-seeking children who were ≤ 18 years on entry into offshore immigration detention on Nauru. *Eleven were separated from ≥ 1 primary relative on Nauru including one unaccompanied minor.*	Mean (SD): 9 (5.7) Range: 3 months – 22 years	38 Male 24 Female	52% of sample held on Nauru for ≥ 4 years	Iran (47%), Iraq (11%), Sri Lanka (10%)	Australia	Physical and mental or neurodevelopmental concerns/conditions; risk and protective factors	80-question health survey, ACEs, R-ACEs	Transferred from Nauru off-shore detention to Australia after protracted detention due to personal or family member medical evacuation.	• 44% (*n* = 27) had a formal diagnosis; pervasive refusal syndrome (*n* = 9, 15%), PTSD (*n* = 8, 13%) and depression (*n* = 8, 13%) • Most frequent symptoms included low mood (*N* = 29, 47%) and sleep difficulties (*n* = 29, 47%) • Suicidal ideation/attempt or self-harm (*n* = 28, 45%). • Formal development screening or assessment only conducted among a minority (*n* = 8, 13%); with 3 of those screened having a neurodevelopmental condition diagnosed.	• 89% (*n* = 55) had a physical health diagnosis; Malnutrition (*n* = 15, 24%), dental disease (*n* = 13, 21%)
Derluyn, 2023 [80]	Analytical cross-sectional study	99 URMs across four detention centres in Tripoli.	Not provided	93 Male 6 Female	Not provided	Eritrea (50.5%), Somalia (26.3%), Nigeria (5%), Sudan (5%)	Libya	Mental health of detained URMs and associations with trauma, flight and daily hardship.	Semi-structured qualitative interviews, scales (SLE, DSSYR, RATS, HSCl-37a)	In detention at time of assessment	• Levels of PTSD (mean 19.99; s.d. = 5.45; range: 8–32), anxiety (mean 19.05; s.d. = 4.82; range: 10–39) and depression (mean 30.93; s.d. = 7.72; range: 16–65) were noted to be relatively high. • Higher social needs were associated with increased anxiety symptoms (β = 0.59; *P* = 0.028) and increased pre-migration (β = 0.10; *P* = 0.061) and peri-migration trauma (β = 0.16; *P* = 0.017) with symptoms of depression. Higher levels of pre-migration trauma were associated with higher post-traumatic stress disorder levels (β = 0.17; *P* = 0.010).	Not examined
Ehntholt, 2018 [6]	Analytical cross-sectional study	35 previously detained unaccompanied asylum-seeking children (UASC).	Age at assessment (years): 19.1 ± 1.12 (16–21) Age in detention (years): 15.8 ± 0.93 (13–17)	21 Male 14 Female	Mean 22.8 ± 21.0; range 4–92 days	Afghanistan (31%), DRC (9%), Iran (9%), Uganda (9%), China (6%), Ivory Coast (6%),	UK	Mental health of UASC in relation to having their ages disputed and being detained.	Diagnostic interviews, self-report measures (SCID-IV, RATS, SLE, DEC-UK)	Interval from detention to assessment (months): 37.5 ± 11.0 (18–74)	• Professionals reported a diagnosis of PTSD developing in 29% (*n* = 10), PTSD exacerbated in 51% (*n* = 18), MDD developing in 23% (*n* = 8) and MDD exacerbated in 40% (*n* = 14). • A total of 3years post-detention, 89% (*n* = 31) met diagnostic criteria for psychiatric disorders and reported high PTSD symptoms. Total SLE score was significantly correlated with RATS total (*r* = 0.39, *p* = 0.026) and intrusion scores (*r* = 0.42, *p* = 0.017). RATS total and sub-scores did not correlate with DEC total score.	Not examined
Essex, 2022 [62]	Cross-sectional study	Estimated total 2454 children detained onshore and 952 total in offshore detention between 2014 Q3 and 2017 Q2. Proportion of asylum seekers/refugees not determined.	Not able to be extracted	Not able to be extracted	Not able to be extracted	Not disclosed	Australia	Quantify health presentation data of children and adolescents in Australian immigration detention and compare rates between onshore and offshore detention.	Australian government’s Quarterly Immigration Detention Health Reports	Not able to be extracted	• Children detained offshore demonstrated significantly higher rates of consultations with mental health professionals, including mental health nurses (z = −1.96; *P* = 0.002), psychologists (z = −2.32; *P* = 0.01), and counsellors (z = −3.41; *P* < 0.001).	• Complaints related to skin (z = −1.97; *P* = 0.05), respiratory (z = −1.96; *P* = 0.05), and urological concerns (z = −2.21; *P* = 0.03) were also significantly higher among children detained offshore.
Hanes, 2019 [59]	Prevalence Cross-sectional study	Estimated total 2454 children detained onshore and 952 total in offshore detention between 2014 Q3 and 2017 Q2. Proportion of asylum seekers/refugees not determined.	Not able to be extracted	61 Male 49 Female	Duration, months, median, IQR At first review: 7 (3-12.5) At end of audit: 13 (5-18.5) Longest duration: 60 months Detention across multiple sites (median 2, IQR 1–3 locations).	Iran (35.5%), Sri Lanka (21.8%), Iraq 15.5%), Afghanistan 10%), Burma (9.1%), Other (8.18%)	Australia	Demographic profile, mental and physical health issues, detention experience and childhood adversity	Refugee health assessments, health records, hospital admissions, R-ACE	74 (67.3%) currently in detention 33 (30%) previously in detention 3 (2.7%) nil detention (via parents)	• Severe acute psychosocial concerns were reported in in children (52/109, 46.3% and 36 of 109 (32.7%) siblings.	• Dietary/Weight concerns (56%) • Dental caries (63.6%) • Vitamin D deficiency- Mild (30–50 nmol/L) (92.4%) • Vitamin D deficiency – Moderate (12.5–29 nmol/L) (7.5%) • Cognitive/Educational/Developmental concerns (52.3%) • Skin disease/Topical infestations (19.2%) • Intestinal infection (14.6%) • Enuresis (13.8%) • Blood disorder (anaemia, haemoglobinopathy) (12.9%) • Somatic symptoms (8.2%) • Tuberculosis (LTBI/active) (4.5%)
Lorek, 2009 [40]	Cross-sectional descriptive study	24 detained child asylum seekers from 16 different families. *18/24 single mother*,* 2/24 single male carer. 2/24 both parents in detention.*	Md = 4.75 years, IQR 0.25–17 years)	12 Male 12 Female	Duration of detention prior to assessment (Median as days (range)) stratified by age band 0–12 months: 43 (15–51) 1–4 years: 50 (18–155) 5–10 years: 32 (11–155) 11–18 years: 57 (43–115)] Total: 11–155 (median 43 days)	Nigeria (*n* = 6), Uganda (*n* = 5), Pakistan (*n* = 3), Jamaica (*n* = 3), Central African Republic (*n* = 2), DRC (*n* = 2),	UK	Mental and physical health of children held within a British immigration detention centre.	Clinical diagnostic interviews, observation (younger children age 3–6) and self-report (older children age 7–11); SCAS, DSRS, R-IES-13), self-report by parents/carers: SDQ and CORE	In detention at time of assessment.	- During the psychological assessment of 11 children, 8 met criteria for psychiatric caseness on the Strengths and Difficulties Questionnaire. All 11 reported symptoms of depression and anxiety. - Sleep problems, somatic complaints, poor appetite, emotional symptoms, and behavioural difficulties were common. - Symptoms of global distress were also reported by all 9 parents.	Age Band: 0–12 months • Inguinal Hernia (*n* = 1) • Eczema (*n* = 1) • Cough (*n* = 1) • Gastroesophageal reflux (*n* = 1) • Loose stool (*n* = 1) • Fever (*n* = 1) 1–4 years • Eczema flare (*n* = 2) • Cough (*n* = 6) • Abdominal pain (*n* = 3) • Vomiting (*n* = 3) • Constipation (*n* = 2) • Pneumonia (*n* = 1) • Fever (*n* = 2) 5–18 years • Eczema (*n* = 1) • Cough (*n* = 2) • Abdominal pains (*n* = 6) • Vomiting (*n* = 1) • Constipation (*n* = 3) • Headaches (*n* = 3) • Ear infection (*n* = 1) • Nosebleed (*n* = 1) • Fever (*n* = 2)
MacLean, 2019 [19]	Analytical cross-sectional study	425 child asylum seekers detained with their mothers.	Range 4–17 years	Not reported but included as a covariate	Detention Mean (SD): 9 (6) days Range: 1–44 days 96% of sample interviewed < 19 days from arrival to detention centre.	Honduras (50%), Guatemala (22%), El Salvador (23%), Nicaragua (2%), Mexico (2%)	USA	Mental health of children held at a US immigration detention centre.	Parent-report SDQ (mothers), PTSD-RI (subset of 150 children aged ≥ 9)	In detention at time of assessment	• Of the 150 children who completed the PTSD-RI, 17% had a probable diagnosis of PTSD. • In all, nearly half (44%) of all children demonstrated at least one emotional or behavioural concern.	Not examined
Mares, 2004 [43]	Cross-sectional prevalence study	20 child asylum seekers from 10 families referred to a child and adolescent mental health service. *7 two parent*,* two sole parent*,* 1 unaccompanied minor*	Range 11 months- 17 years	14 male 6 female	Median detention at initial contact: 15 months (IQR 12–18 months)	Not stated	Australia	Mental health of families held at an immigration detention centre.	Comprehensive clinical assessment through multiple interviews	Held in a remote detention centre at time of assessment	Of 10 children < 5 years • 50% had delays in language and social development and/or emotional and behavioural dysregulation • 80% had emotional or developmental disturbance Of 10 children aged 6–17 years • 100% met criteria for both PTSD and Major depression with suicidal ideation. • 80% including three pre- adolescents, had made significant attempts at self- harm. • 70% had symptoms of an anxiety disorder. • 80% had a developmental delay of emotional disturbance • 100% of children had at least one parent with a psychiatric illness.	Of 10 children aged 6–17 years - 50% reported severe somatic symptoms (headaches and abdominal pains)
Mares, 2016 [32]	Analytical cross-sectional study using retrospective data	191 child asylum seekers detained on Christmas Island detention centre. *70 in single-parent families; 56 in two parents; 15 of 12–17 yo were unaccompanied.*	Range 0–17; Mean/SD: 7.64 (4.89)	Not reported	Mean detention: 209.5 days (7 months), SD: 62.36 days	Iran, Sri Lanka, Iraq, Afghanistan, Vietnma, Somalia, Syria	Australia	Mental health of asylum-seeking children during prolonged detention.	K10 (adolescents aged 12–17, *n* = 35); SDQ (children aged 3–17, *n* = 70), responses from parents about infants’ wellbeing (*n* = 48)	Held in detention at time of assessment	• Severe co-morbid depression and anxiety in 85.7% of teenagers. • 75.7% of children had a high probability of psychiatric disorder, with lower conduct and hyperactivity scores than clinic populations. • 67% percent of parents had concerns about the impact of detention on infant’s emotional or mental health and 23% on their development. • Correlations were not found between length of detention and severity of psychological distress in children.	Not examined
Rothe 2003 [81]	Cross-sectional prevalence study	74 detained child asylum seekers, presenting to a psychiatric clinic at Guantanamo Bay detention centre *19 adolescents were unaccompanied.*	Mean age: 15.5 years SD = 0.7)	47 Male 27 female	Not provided	Cuba	USA	Mental health and experiences of detained adolescent asylum seekers.	PTSD-RI, Medical interview	Held in detention at time of assessment	• All 74 adolescents had PTSD-RI scores ranging from severe to very severe • 94% of the adolescent boys and 96% of the adolescent girls scoring in the very severe (highest) category of traumatic stress symptoms. • 64% of the adolescent boys and 89% of the adolescent girls cited that life in the detention camp was their worst moment since leaving Cuba.	Not examined
Rothe 2002 [83]	Analytical cross-sectional study	87 refugee children previously in a detention camp. − 1*1 adolescents left their mother behind.* *−18 adolescents left their father behind.*	14.9 years (Range 6–17)	50 male 37 female	Mean 6–8 months	Cuba	USA	Self-reported symptoms of PTSD; teachers’ assessments of behavioural problems.	PTSD-RI, CBCL-TRF	Released from Guantanamo Bay detention 4–6 months ago	• Up to 6-months post leaving detention 57% of children met diagnostic criteria for PTSD; 25% had severe PTSD. • Most common symptom clusters: avoidance (67%), regressive behaviours (64%), re-experiencing the traumatic events (60%), somatic symptoms (52%), and hyper-arousal (51%).	Not examined
Sidamon-Eristoff, 2022 [82]	Analytical cross-sectional study	84 previously detained child asylum seekers - *Nil unaccompanied*.	Mean age = 7.78 ± 4.18; range: 1–17 years_	38 male 46 female	Mean detention length ± SD: 7.31 ± 7.15 days	Honduras (63.6%), El Salvador (23.8%), Mexico 10.7%), Guatemala (9.5%), Nicaragua (2.4%)	USA	PTSD symptomatology, rates of traumatic events, detention experience	PTSD-RI	All families were interviewed within 2 days of release from immigration detention.	77 children completed the screening for PTSD: • A total of 97.4% of children experienced at least one premigration traumatic event. • 37.66% (*n* = 29) of children met criteria for DSM-5 category B intrusion symptoms, 27.27% (*n* = 21) for category C avoidance symptoms, 22.08% (*n* = 17) for category D negative alterations in cognition and mood symptoms, and 18.18% (*n* = 14) met criteria for category E arousal and reactivity symptoms. • 27.27% (*n* = 21) of children met only one symptom criterion of PTSD with 72.7% meeting 2 to 4 of symptom criteria. • PTSD symptom severity was most strongly predicted by premigration trauma (B = 3.76,t = 6.106, *p* < 0.0001), followed by duration of parent–child separation (B = 2.03, t = 1.867, *p* = 0.066) and an interaction between premigration trauma, length of detention, and length of parent–child separation (B = 0.202,t = 1.814, *p* = 0.074).-	Not examined
Steel, 2004 [79]	Cross-sectional prevalence study	22 child asylum seekers from 10 families held in detention for more than 2 years. -*Nil unaccompanied*	Range 3–19 years	13 males 9 females	Mean detention: 28months (IQR: 24–32 months)	Not disclosed but all same ethnic background	Australia	Psychiatric status of families held for a protracted period in immigration detention	Structured psychiatric interviews. DECL, DSCL, K-SADS-PL.	Held in remote detention facility at time of assessment	Of the 20 children assessed: • 10% had Major depressive disorder, 5% PTSD, 10% separation anxiety disorder prior to their detention. • At assessment after mean detention 28 months: 95% had Major depressive disorder, 50% PTSD, 50% separation anxiety disorder, 45% oppositional defiant disorder, 20% enuresis, 55% suicidal ideation and 25% had self-harm.	Not examined
Tosif, 2023 [61]	Analytical Cross-sectional study	277 child asylum seekers held in detention or born to parents who had been detained in Australia/Australian territories (*n* = 198) or Nauru/Manus Island (*n* = 79) *− 58 children had been separated from ≥ 1 parent in detention*	Median age (IQR): 4.2 (0.7–7.7);	145 males 132 females	Median duration detention: 12 months (IQR 5–19 months)	Iran (42%), Malaysia (7%), Sri Lanka(6%), Iraq (4%), India (4%),	Australia	The physical and mental health of children and families who had experienced immigration detention.	Medical records.	239 had directly experienced held detention, including 79 children from families detained on Nauru or Manus Island (Papua New Guinea), with the remaining 38 children being born into families after release from detention (time since release from community unable to be ascertained).	• 62% of children diagnosed with a mental health diagnosis: PTSD (30%), anxiety (44%), depression (32%), behaviour disorder (40%), attachment disorders (25%). • 52% of children had psychiatric symptoms: Nightmares (27%), Sleep Difficulties (43%), School refusal (13%), somatic complaints (18%), Self-harm (10%)	• Nutritional deficiency (60%); Low Vitamin D (51%), Iron deficiency without anaemia excluding thalassaemia (22%) low B12 (2%), iron deficiency anaemia (1%), low zinc (1%) • Infectious diseases (28%); Latent TB (21%), Strongyloidiasis (3%), Helicobacter pylori stool antigen positive (3%) • Developmental concerns (75%); parent concern regarding development (38%), learning difficulty e.g. language delay or concentration difficulty (30%), vision concerns (20%), physical disability (12%), hearing concerns (11%), autism (10%), intellectual disability 9%). • Other medical issues (96%); dental concerns (43%), feeding difficulties (40%), constipation (21%), secondary nocturnal enuresis (19%), headaches (10%), encopresis (5%)
Zwi, 2017 [26]	Analytical cross-sectional study	48 child asylum detained since arrival compared with 38 child refugees who had never been detained and living in community *Unaccompanied (%) unable to be determined.*	Age range 4–15; mean age 8.4.	Not provided for detained sample	Mean detention: 221 days (7 months) (IQR 3–13 months)	Not provided for detained sample	Australia	Social-emotional well-being of community-based and detained ASR	Parent-report SDQ	The detention sample had been living in detention for a mean of 221 days at the time of assessment. The community sample had been living in Australia for 325 days, with no time in detention.	• Children in the detention group had significantly higher SDQ total difficulties scores than refugee children in the community group (*P* < 0.0001) indicating clinically significant social, emotional and behavioural difficulties. • Compared to Australian norms, the detention group had significantly higher scores (*P* < 0.001) for all except Pro-social scores for the 4–6 and 7–15 years age group. This was not the case for the community group.	Not examined

*ACEs* Adverse Childhood Experiences, *CBCL-TRF* Child Behavioural Check List- Teacher Form, *CORE* Clinical Outcomes in Routine Evaluation, *CYP* Child and Young People, *DEC-UK*Detention Experiences Checklist-UK version, *DECL* Detention Experiences Checklist, *DSCL*Detention Symptoms Checklist, *DSRS* Birleson Depression Self-Rating Scale, *DSSYR* Daily Stressors Scale for Young Refugees, *HSCL-37 A* Hopkins Symptom Checklist-37 A, *K-SADS-PL* Schedule for Affective Disorders and Schizophrenia for School-Age Children – Present and Lifetime Version, *PTSD-RI* Post-traumatic Stress Disorder Reaction Index, *R-ACE* Refugee Adverse Childhood Experiences, *R-IES-13* Revised Impact of Event Scale-13 (for PTSD), *RATS* Reactions of Adolescents to Traumatic Stress questionnaire, *SCAS* Spence Children’s Anxiety Scale, *SCID-IV* Structured Clinical Interview for DSM-IV Axis I Disorders, *SCID-IV* Structured Clinical Interview for DSM-IV, *SDQ* Strengths and Difficulties Questionnaire, *SLE* Stressful Live Events questionnaire, *UASC* Unaccompanied Asylum-Seeking Children, *UCLA PTSD-RI*UCLA Post-traumatic Stress Disorder Reaction Index, *URM* Undocumented Refugee Minor

### Detention of children and implications for physical and mental health – global trends

Table 1 summarises the characteristics and key findings of the impacts of immigration detention on the health of child asylum seekers and refugees in the 15 included studies. The analysis reveals systemic patterns of harm exacerbated by detention duration, family separation, and dehumanising conditions, while contextual variations highlight the role of policy frameworks in shaping outcomes.

#### Mental health

##### Australia

Australia’s mandatory detention policies, particularly for offshore processing of asylum-seeking children, resulted in the most severe and well-documented harm.

Mares and Jureidini (2004) studied 20 children who had been detained and were assessed over a 15-month period by Australian clinicians. There were very high levels of psychopathology among child asylum seekers; 100% of the 10 children aged 6–17 years met diagnostic criteria for PTSD and MDD with suicidal ideation. Eight children, including three pre-adolescents, had made significant self-harm attempts; seven had symptoms of an anxiety disorder (panic disorder, generalised anxiety disorder, separation anxiety), and half reported persistent severe somatic symptoms, particularly headaches and abdominal pain. The majority (80%) of preschool-age children demonstrated developmental regression or emotional disturbances, and all children reported extreme boredom, anxiety about falling behind in their schoolwork and shame about knowing less than their age-appropriate peers [43].

Similarly, all 20 children in Steel et al. (2004) [79] were diagnosed with at least one psychiatric disorder, and most (*n* = 16, 80%) were diagnosed with multiple disorders. Prior to detention, there were low levels of psychopathology, with 10% having MDD, 5% PTSD, and 10% having separation anxiety. At assessment and after a mean detention of 28 months, 95% of the children had developed MDD, 50% PTSD, 50% separation anxiety disorder, 45% oppositional defiant disorder, 29% enuresis, 55% suicidal ideation and 25% self-harm, representing a tenfold increase compared to the number of diagnoses recorded before detention. There were 52 psychiatric disorders among 20 children, a rate noted to be many times higher than in the host child population [85]. In some cases, symptoms of PTSD were directly related to experiences in detention, with children describing nightmares about being hit by officers; physical assault by detention officers was alleged by a third of the children (*n* = 7, 37%). The majority were regularly distressed by sudden and upsetting memories about detention, intrusive images of events that had occurred in detention, and feelings of sadness and hopelessness about currently being in detention. The children reported extreme distress associated with the fear of being repatriated (*n* = 16, 84%) and rated being called by a number rather than their name as a serious problem (*n* = 9, 47%) [79].

Amarasena et al. (2023) sampled 62 ASR children who had been transferred from Nauru off-shore^1^ detention to Australia owing to personal or familial medical evacuation. Thirty-two (59%) had experienced detention for four or more years, 13 (24%) spent 0–23 months, and 9 (17%) for 24–47 months. Following any duration of detention, there were significant incidences of mental disorders (*n* = 27, 44%), predominantly including pervasive refusal syndrome (*n* = 9, 15%), (PTSD) (*n* = 8, 13%), and MDD (*n* = 8, 13%). In total, 49 children (79%) demonstrated at least one mental health concern, with low mood (*n* = 29, 47%) and sleep difficulties (*n* = 29, 47%) identified as prevalent symptoms. Nearly half of the participants (*n* = 28, 45%) indicated experiences of suicidal ideation or attempts, as well as self-harm. It was noted that mental health concerns were more likely among those who: were school-aged (*p* = 0.001); had been held on Nauru for ≥ 1 year (*p* = 0.01); originated from the Eastern Mediterranean region (*p* < 0.05); or had witnessed trauma (*p* < 0.05) or had exposure to ≥ 4 refugee-specific adverse childhood experiences (*p* < 0.05) [60].

Tosif et al. (2024) [61] sampled 277 children, of whom 239 had directly experienced held detention, including 79 children from families detained on Nauru or Manus Island (Papua New Guinea), with the remaining 38 children being born into families after release from detention. Children who were detained at Nauru or Manus Island (Median = 51 months, IQR 29–60) experienced significantly longer periods of detention when compared to those confined exclusively within Australian territories (Median = 7 months, IQR: 4–16) (*p* < 0.001). At the time of evaluation, pronounced rates of developmental delays were observed among the sample of children, with a median age of 5.1 years (IQR: 1.8–8.3 years) upon entering detention; these children were frequently held in multiple detention centres (Median = 3 centres, range 1–8). Following a median detention duration of 12 months (IQR: 5–19 months), 75% (*n* = 207) exhibited developmental concerns, with 38% attributed to parental apprehensions regarding development and 20% related to learning difficulties. This cohort included 10% (*n* = 27) diagnosed with autism spectrum disorder and 9% (*n* = 26) with intellectual disabilities. Furthermore, mental health issues were noted in 62% (*n* = 171) of the children, encompassing anxiety, depression, and behavioural disturbances, with an additional 54% (*n* = 150) having parents suffering from mental illness. Notably, during or subsequent to their detention, 12% (*n* = 33) of the children encountered parental separation, leading to family breakdown.

Hanes et al. [59] assessed 110 asylum-seeking children (Mean/SD age: 6 ± 4.72 years) with high detention rates (107/110, 92%) at an Australian paediatric refugee health service. The children exhibited a comparable age profile to that of Mares & Jureidini [43] and Amarasena et al. [60]. Similarly, there were elevated rates of cognitive, educational, and developmental concerns (52.3%) among the sample of 110 asylum-seeking children (Mean age: 6 years), of whom 67.3% (*n* = 74) were residing in detention at the time of assessment at a paediatric health clinic in Australia. ASR children were also observed to present complex and severe psychosocial concerns, which were reported in nearly half of the cohort (52/109, 46%), attributed to prolonged exposure to detention and lack of access to necessary services.

##### **Libya**

Derluyn et al. (2023) highlighted the compounding effects of detention on 99 unaccompanied minors in Libya, where systemic neglect exacerbated pre-migration vulnerabilities. Reports of daily hardship within the confines of immigration detention in Libya ranged between 40% and 95% for insufficient fulfilment of basic needs and between 27% and 80% for social needs [80]. Higher unfulfilled social needs as a consequence of being in immigration detention were significantly associated with increased anxiety symptoms. Immigration detention was noted as a specific traumatic event in this study, and participants reported high rates of trauma across all events and time points, with a mean of 1.77 (SD = 1.93; range: 0–8) traumatic events pre-migration, a mean of 1.59 (SD = 1.86; range: 0–7) traumatic events in transit journey to Libya, and a mean of 4.21 (SD = 2.02; range: 0–8) traumatic events since arrival at the Libyan detention facility, highlighting that rates of traumatic events were highest after arrival in Libya and immigration detention. Furthermore, levels of PTSD (M = 19.99; SD = 5.45; range: 8–32; max score: 32), anxiety (M = 19.05; SD = 4.82; range: 10–39; max score: 40) and depression (M = 30.93; SD = 7.72; range: 16–56; max score: 56) were relatively high in this sample, with increased traumatic events in transit journey to Libya (β = 0.16; *P* = 0.017), including detention, resulting in greater symptoms of depression. Similarly, greater exposure to pre-migration trauma was significantly associated with higher levels of PTSD (β = 0.17; *P* = 0.010) [80].

##### United Kingdom

UK studies revealed acute and long-term mental health consequences, even for children detained briefly.

In Lorek et al. (2009) [40], a total of 24 detained children (Median age = 4.75 years, Range: 3 months −17 years) held in a UK detention centre were assessed by independent clinicians in the context of a legal challenge to their detention. After a median detention duration of 43 days, psychological assessments of 11 of the children demonstrated that all (*n* = 11/24, 45.8%) had begun to display acute symptoms of depression and anxiety. A total of 11 children (aged 3–11 years) completed psychological assessments; of the six older children (7–11 years) who completed the self-report questionnaire, five children had a likely diagnosis of clinical depression, and four experienced significant levels of anxiety. Of those scoring in the clinical range for anxiety, all four were above the cut-off for separation anxiety, three for physical injury fears, three for generalised anxiety, two for panic, and one for obsessive-compulsive symptoms. One child also reported experiencing the re-emergence of PTSD symptoms related to previous war experiences. Of the 20 children seen by a paediatrician, several had missed preventative health and medical appointments, and all mothers of the eight children aged 1–4 years seen by a paediatrician or psychologist raised concerns about their children’s development or behaviour [40].

In Ehntholt et al. (2018) [6], a team of clinicians interviewed a group of 35 formerly detained unaccompanied child asylum-seekers who were seeking legal recourse after British authorities had disputed their age, resulting in their detention. The mean duration of detention was 22.8 ± 21.0 days, with 54% having been previously detained before their UK detention experience and 49% with a history of torture. Three years post-detention, longitudinal harms were demonstrated, with 89% of the sample (*n* = 31) meeting diagnostic criteria for PTSD, MDD or both (34%, 9% and 46%, respectively). Additionally, 80% (*n* = 28) of all participants fell within the ‘very high’ category for PTSD symptoms [86]. Based on the clinician’s assessment, 29% (*n* = 10) of the participants were likely to have developed PTSD during their detention, while for 51% (*n* = 18) of participants, the detention exacerbated existing PTSD. Additionally, MDD likely developed during detention in 23% (*n* = 8) and was likely exacerbated by detention in 40% (*n* = 14) of the sample as a consequence of detention.

##### USA

US studies demonstrated divergent outcomes influenced by detention duration, family separation, and pre-migration trauma.

Rothe et al. (2003) evaluated 74 Cuban adolescents who had presented to the psychiatric clinic for help during their time at the Guantanamo Bay detention centre [81]. There were low rates of pre-existing psychiatric disorders (14% of boys and 11% of girls). Cuban adolescents held in immigration detention exhibited elevated rates of severe traumatic stress (94% of boys and 96% of girls), including nightmares (74% boys, 96% girls), sleep disturbances (85% boys, 96% girls), appetite disturbances (48% boys, 51% girls) and enuresis/encopresis (45% boys, 48% girls) [81].

Following an average duration of 6 to 8 months in the Guantanamo Bay detention facility, a cohort of 87 formerly detained children and adolescents (Mean age = 14.9 years, IQR = 6–17 years) was assessed 4 to 6 months subsequent to their release. Among these children, 50 individuals (57%) received positive scores for PTSD on the PTSD Reaction Index (PTSDRI), with 28 children (32%) exhibiting moderate PTSD symptoms, and 22 children (25%) displaying severe PTSD symptoms. Although age emerged as the sole significant predictor of trauma scores, the experience of witnessing violence within the detention facility approached statistical significance. Alongside the fear of dying at sea, witnessing violence constituted the only other significant predictor for withdrawal symptoms as measured by the Child Behaviour Checklist Teacher Report Form (CBCL-TRF). Educators in the study noted mild difficulties in children, but overall academic and social functioning remained largely unaffected [83].

MacLean et al. (2019) [19] investigated the mental health of children in immigration detention by interviewing 425 mothers regarding their eldest child, aged 4 to 17 years (M = 10 ± 4 years). Additionally, a subset of 150 children aged 9 years or older was surveyed. Among the 425 children, elevated scores were observed, indicating greater difficulties in emotional problems (32%), peer problems (14%), and total difficulties (10%). Notably, 44% of all children exhibited at least one emotional or behavioural concern. Younger children, aged 4 to 8 years, demonstrated more pronounced difficulties relating to conduct, hyperactivity, and total difficulties (all *p* < 0.001) compared to their older counterparts. Among the subset of 150 children aged 9 years or older, 17% displayed probable PTSD, with results not varying significantly by age, gender, maternal separation, or country of origin. Although 96.7% of the sample was interviewed within 19 days of arrival at the detention centre, the prevalence of emotional, peer, and total difficulties remained high, likely attributable to pre- and peri-migration experiences, ongoing detention, and a traumatic background, all of which can adversely affect developmental outcomes [19].

Sidamon-Eristoff et al. (2022) [82] interviewed the parents of 84 Central-American and Mexican children (55% female; M = 7.78; ±4.18 years) of families released from US-based immigration detention after a mean duration of 7.31 ± 7.15 days. Families were interviewed within two days of their release from detention, and children who had been separated from their parents at the United States–Mexico border had been reunited with their families by the time the interviews were conducted. Only a small number of children (*n* = 4; 4.9%) were detained for more than 20 days. A substantial incidence of trauma exposure was observed among the population of 77 children from whom data was collected, with an aggregate of 97.4% (*n* = 75) of these children having encountered at least one premigration traumatic event (M = 3.29 ± 2.13, range = 0–10 events). However, only a small subset (*n* = 5; 6.49%) of the children met criteria for PTSD, whereas four (5.19%) presented with sub-threshold symptoms. This suggests that brief detention and expedited reunification may mitigate the risk of severe psychopathology [82].

#### Physical health

Data pertaining to physical health was reported across six studies [40, 43, 59–62], with only one study [40] conducted outside Australia.

Mares and Jureidini [43] observed that, following a median duratiofvn of detention of 15 months among a sample of ten children aged 6–17 years, 50% reported experiencing significant somatic symptoms, predominantly headaches and abdominal discomfort.

The physical health issues identified by Tosif et al. [61] encompassed dental problems (*n* = 118; 42.5%), constipation (*n* = 57; 20.6%), and secondary nocturnal enuresis (*n* = 54; 19.5%). Additionally, feeding difficulties, including inadequate intake, fussiness, and food refusal, were frequently reported (*n* = 111; 40%), particularly among infants from families detained on Nauru or Manus Island (51%, *n* = 40/79) and among infants born in detention within Australian territory (52%, *n* = 16/31) [61]. Collectively, 60% of the entire sample (*N* = 277) of children exhibited nutritional deficiencies, with 51% experiencing low vitamin D levels and 22% presenting with iron deficiency.

Similar to Tosif et al., detention was across multiple sites in Hanes et al. [59] (Md = 2, IQR 1–3) with a similar median duration of seven months (IQR 3–12.5 months) at the time of initial review. High rates of family separation (91/108, 84%) and interrupted education (41/46, 89%) were noted. At the time of hospital interaction, point prevalence estimates for physical health concerns included malnutrition (61/109, 56%), dental caries (59/110, 54%) and vitamin D deficiency (level < 50 nmol/L; 53/108, 49%). Other physical health diagnoses included dermatological conditions (21/109, 19%), enuresis (16/109, 15%) and haematological issues, including anaemia and/or haemoglobinopathy (14/108, 13%).

Essex et al. (2022) [62] compared the health and healthcare needs of children and adolescents in Australian onshore and offshore detention with those observed in the Australian community. Physical complaints related to skin (z = −1.97; *P* = 0.05), respiratory issues (z = −1.96; *P* = 0.05), and urological issues (z = −2.21; *P* = 0.03) were significantly higher among children detained offshore. Additionally, prescription rates for medications were generally higher offshore, with significant differences noted for three drug classes: penicillins (z = −3.10; *P* < 0.001), antihistamines (z = −3.66; *P* < 0.001), and expectorants (z = −3.56; *P* < 0.001). Between 9% and 26% of children were prescribed penicillin offshore per quarter, which was, on average, approximately three times higher than the prescription rates observed onshore. The authors noted that 1.8% of Australians aged 0–17 years were prescribed antidepressants between 2017 and 2018, compared with up to 8% of the ASR population onshore and 5% of the population offshore [62].

Similarly, physical health issues affected 89% (*n* = 55) of the children in the Amarasena et al. (2003) study who had experienced protracted off-shore detention in Nauru, notably malnutrition (*n* = 15, 24%) and dental disease (*n* = 13, 21%) [60].

In Lorek et al. [40], following a median duration of 32 days in the United Kingdom, somatic complaints were reported among the child asylum-seeking detainees, primarily comprising headaches and abdominal pain, which developed in 10 of the 11 children.

#### Immigration detention systemic risk factors

##### Duration of detention

Protracted detention periods, with a median duration of 12 months, was associated with developmental concerns in 75% of children and parental mental illness in 62% [61]. Parental mental health issues were significant, affecting 54% of children, including 86% of those within the offshore Nauru/Manus Island cohort. Parental relationship dissolution occurred in 12% of families during or after detention.

##### Adverse childhood experiences

A moderate dose-response relationship emerged between the duration of detention and the frequency of distressing detention-related experiences [6]. Common detention-related traumatic exposures included body searches (90%, *n* = 30) and sudden removal to detention without warning (72%, *n* = 24) [6]. Dehumanising practices, such as identifying children by numbers instead of names, caused distress in 47% of cases, while 84% reported an additional fear of repatriation [79].

Cumulative trauma exposure was prevalent across studies. In Hanes et al., multiple occurrences of Refugee Adverse Childhood Experiences (R-ACEs) were identified within a sample of 110 children, of whom 67.3% were in detention at the time of the review. Every child experienced ≥ 3 R-ACEs (Median = 4, IQR = 4–5). Notably, 84% of the children had encountered family separation, with 47% being separated from their families at the time of assessment, and 89.1% had faced interruptions in their education. Furthermore, there were elevated rates of parental mental illness, affecting 53.6% of the sample [59]. Similarly, Amarasena et al. observed that 58 children and adolescents (94%) experienced at least one Adverse Childhood Experience (ACE) in their lifetime. This assessment covered pre-, peri-, and post-migration contexts. Among them, 13 (21%) faced ≥ 4 ACEs, with a median of 2 and an IQR of 1 to 3. Furthermore, 25 (40%) reported exposure to at least one type of abuse or neglect, including 15 individuals (24%) who experienced physical, sexual, or emotional abuse. Additionally, 39 (63%) reported witnessing traumatic events [60].

##### Family separation

Forcible separation from mothers was associated with significantly higher rates of emotional problems (49% vs. 29%, *p* = 0.003) and total difficulties (15% vs. 9%, *p* = 0.015) compared to detained children who had never been separated [19].

It is noteworthy that, despite 97.4% (*n* = 75) of the sample in Sidamon-Eristoff [82] having experienced at least one premigration traumatic event (M = 3.29 ± 2.13, range = 0–10 events), only a minor subset (*n* = 5; 6.49%) of the children met the criteria for PTSD following a brief detention period of 7.31 days. The severity of PTSD symptoms was most significantly predicted by premigration trauma (*β* = 3.76,t = 6.106, *p* < 0.0001), followed by the duration of parent-child separation ( *β*= 2.03, t = 1.867, *p* = 0.066) and an interaction involving premigration trauma, length of detention, and duration of parent-child separation (*β* = 0.202,t = 1.814, *p* = 0.074), all of which approached statistical significance.

##### Comparative burden vs. non-detained peers

Children in detention had increased utilisation of mental health services: those held in offshore detention exhibited markedly higher rates of consultations with mental health nurses (z = −1.96; *P* = 0.002), psychologists (z = −2.32; *P* = 0.01), and counsellors (z = −3.41; *P* < 0.001) in comparison to their peers in the Australian community [62].

Zwi et al. (2017) compared 38 resettled refugee children who were not detained, with 48 detained asylum-seeker children. The detained group had an average detention length of 7 months (3–13 months), and both groups had a mean age of 8.4 years. Detained children had significantly higher total difficulties scores on the Strengths and Difficulties Questionnaire (SDQ) than those in the community group. The most notable differences were in emotional problems, hyperactivity, and conduct disorder subscales. The detention group’s average SDQ score was 12 points higher than the community group and exceeded Australian norms for the 4–6 and 7–15 year age groups [76].

### Meta-analysis of children’s SDQ scales

The Strengths and Difficulties Questionnaire (SDQ) was used in four studies in this review, thus enabling a meta-analysis [19, 32, 40, 76]. The common effect size was calculated as a weighted mean and 95% confidence interval (CI) for each study outcome, SDQ_Emotion, SDQ_Hyperactivity, SDQ_Conduct, SDQ_Peer_problem, SDQ_Total and SDQ_Pro_social. To assess publication bias, funnel plots were drawn, and Egger’s and Begg’s tests [87] were conducted to evaluate small study bias. In the case of evidence for publication bias, a meta-trim was used, according to Duval and Tweedie 2000 [88]. Due to the small number of studies, we were not able to carry out meta-regression or subgroup analyses. In addition, to assess the effect of individual studies on effect size, sensitivity analyses were conducted using the leave-one-out analysis.

#### Common effect size

**Fig. 2:**
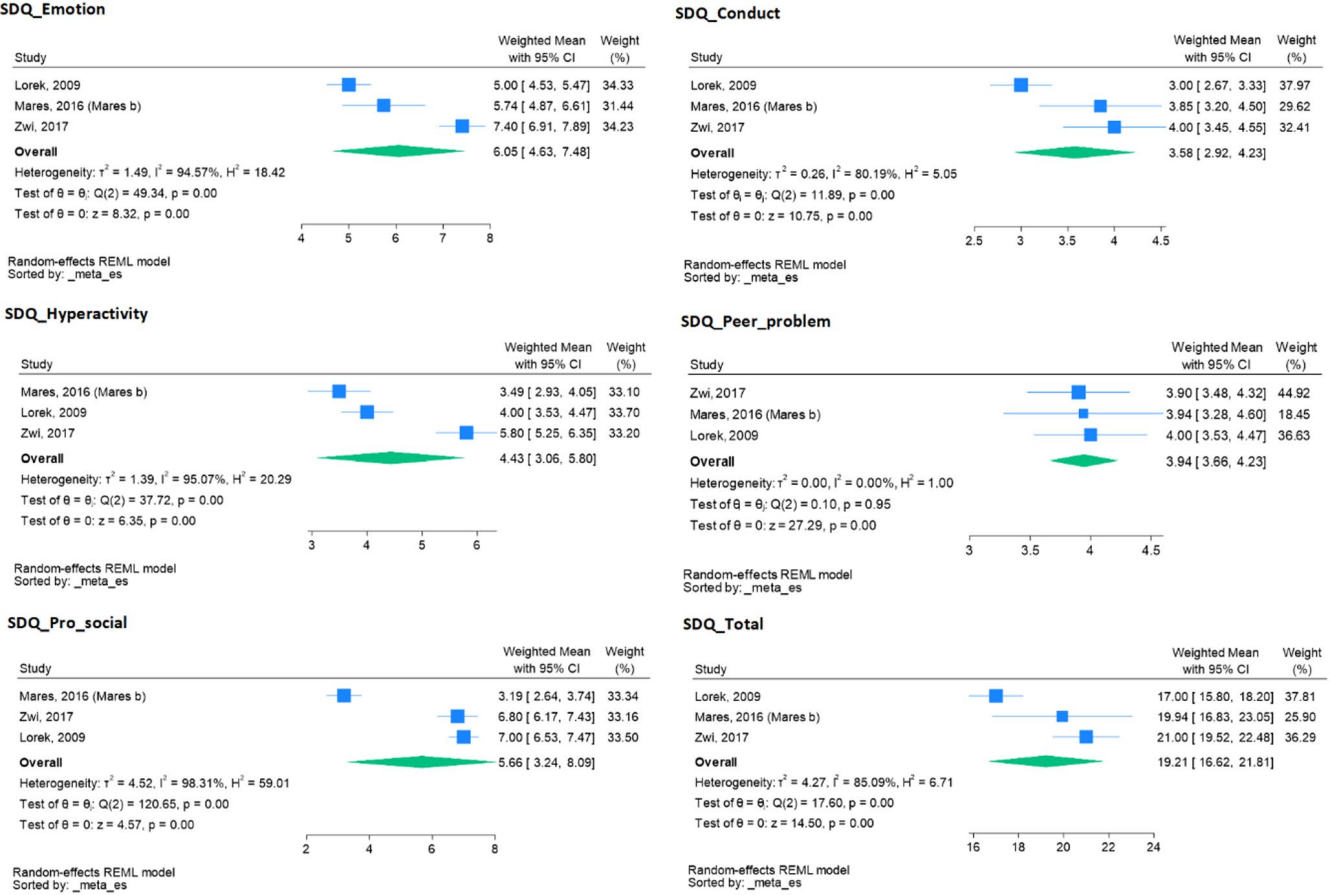
Forest plot for the common effect size for the children’s mental health outcomes

In the mixed model meta-analysis, the forest plot depicted common mean scores and their respective 95% confidence intervals (CI) as follows: SDQ_Emotion (5.40, 95% CI: 3.79–7.01), SDQ_Conduct (2.93, 95% CI: 1.53–4.32), SDQ_Hyperactivity (2.58, 95% CI: 0.88–4.27), SDQ_Peer_problem (3.34, 95% CI: 2.17–4.51), SDQ_ Total (16.63, 95% CI: 11.19–22.07), and SDQ_Pro_social (6.60, 95% CI: 4.10–9.10). See Fig. 2.

#### Heterogeneity of studies

Very high heterogeneity between studies was detected for all outcome measures, with I-squared values ranging from 97.03% for SDQ_Hyperactivity to 99.37% for SDQ_Pro_social. Therefore, a cautious interpretation of the common effect sizes (Fig. 2) is advised.

#### Publication bias

The assessment for publication bias revealed no significant evidence of bias across all outcome measures except for SDQ_Conduct and SDQ_Peer_problem, where Egger’s test indicated potential bias (*p* = 0.011; *p* = 0.012 respectively) but Begg’s test was not significant (*p* = 0.999 for both).

Although the funnel plots for SDQ_Emotion, SDQ_Hyperactivity, and SDQ_Pro_social appeared symmetric acknowledging the limitation of a small number of studies, those for SDQ_Conduct, SDQ_Peer_problem, and SDQ_Total appeared non-symmetric, indicating potential publication bias (see Figure S2). To further test for publication bias, a trim and fill analysis was conducted. This analysis suggested no need for imputing additional studies to compensate for bias for SDQ_Emotion, SDQ_Hyperactivity, and SDQ_Pro_social. However, for SDQ_Conduct, SDQ_Peer_problem, and SDQ_Total, the trim and fill analysis resulted in revised effect sizes of 2.58 (95% CI: 1.29–3.86), 3.12 (95% CI: 2.12–4.12), and 15.16 (95% CI: 10.09–20.24), respectively.

#### Leave-one-out analysis for the outlier detection

Sensitivity analysis, performed through leave-one-out analysis for all outcome measures, revealed no outliers in the estimation of effect sizes. This was substantiated by the consistent intersection of the 95% confidence intervals with the common effect size, except for SDQ_Peer_problem, where the study conducted by MacLean (2019) was identified as an outlier and removed from the analysis due to 96% of the sample having been held in detention for ≤19 days [19]. Upon removal of this study, the effect size increased to (3.94, 95% CI: 3.66–4.23) (refer to Figure S5).

## Discussion

In this systematic review and meta-analysis, 15 studies consisting of 4,890 children were synthesised to document the types and prevalence of physical and mental health conditions and associated risk factors among ASR children with an experience of immigration detention. The SDQ was utilised to meta-analytically compare behavioural and emotional difficulties for 239 detained ASR children across three studies.

### Systematic review main findings

The main findings indicated high levels of psychological distress among detained child ASR, including high rates of depression, anxiety, PTSD and suicidal ideation, alongside developmental issues and social and behavioural difficulties. Parent-child separations in the course of immigration detention aggravated children’s stress responses via disruptions in attachment and secure parental relationships. Although evidence on physical health was limited, detained children showed higher rates of malnutrition, vitamin deficiencies, dental disease, and somatic complaints.

In Australia, all included studies [32, 43, 59–62, 76, 79] highlighted the profound adverse impact of immigration detention on the health and well-being of children seeking asylum. Prolonged detention was noted to be associated with considerably greater physical, mental and neurodevelopmental health issues, including malnutrition, dental disease and vitamin D deficiency, along with developmental delays, PTSD, MDD, and anxiety [27, 43, 59–61, 79]. These studies also reveal high rates of suicidal ideation, self-harm, and complex psychosocial issues among these children [43, 59–61, 79]. Compared to non-detained refugee children, detained children exhibited significantly elevated emotional, behavioural, and conduct problems [62, 76], had higher rates of accessing healthcare and psychological support services, and being prescribed medications, with these rates being even more pronounced for children detained offshore [62].

Associations between social-emotional distress (SDQ total score) and detention duration were found in most studies [31, 52, 53, 55, 56, 89]; however, Zwi et al. (2017) and Mares (2016) reported no clear correlation, potentially due to small sample sizes [76] or incomplete data [32]. Children residing in immigration detention facilities encounter social, emotional, and behavioural challenges at elevated rates compared to their community counterparts [27], including delays in developmental and behavioural milestones [40]. The adverse ramifications of detention exposure are more pervasive and sustained when detention is protracted and/or involves multiple relocations to other detention sites, evidenced by high rates of physical and mental health disorders, including PTSD and long-term relationship difficulties [57, 90–93]. Moreover, children in detention are frequently exposed to violence and acts of self-harm and are surrounded by adults with mental illness. Parents also feel unable to care for or support their children, compounding their sense of hopelessness and lack of control [52, 53, 79, 89, 94]. Underscoring these findings, no time spent in immigration detention has been quantified as ‘safe’ for children [95].

Steel et al. (2004) observed a tenfold increase in psychiatric disorders following detention [79], with prevalence rates exceeding those in refugee children who had not been detained [96–98]. Similarly, PTSD rates among detained Cuban adolescents rose from 11% to over 90% post-detention, with this corresponding to a 6.7-fold and 8.7-fold increase for boys and girls, respectively [81]. The 17% prevalence of probable PTSD found by MacLean et al. (2019) [19] in asylum-seeking children in an immigration detention centre in the USA aligns with the reported rate of 20% among children in immigration detention in the UK [99], yet is far higher than the lifetime prevalence of PTSD among adolescents in the general US population of 4.7% [100]. Additionally, the high rates of clinically significant emotional (32%) and peer problems (14%) seen in MacLean et al. (2019) were consistent with the high rates of “abnormal” scores for these subscales in Zwi et al.’s (2017) sample of child ASR detainees in Australia (21%).

Long-term effects were seen in Ethntholt et al. (2018) [6] with 89% unaccompanied child asylum seekers meeting the criteria for psychiatric disorders three years post-release from detention (mean duration of 22.8 ± 21.0 days). This observation suggests the potential influence of additional post-migration factors on the psychological well-being of children. Of note, 54% of the sample had previously been detained, and 49% had a history of trauma, with the possibility of pre- and peri-migration trauma serving as contributing factors. Similarly, nearly all children (97.4%) in Sidamon-Eristoff’s (2022) [82] sample had experienced pre-migration trauma, exceeding US national rates of 60–62% [100, 101].

The UN Convention on the Rights of the Child [33, 34] dictates that children in detention should have the right to their best interests taken as a primary consideration (Article 3), the rights and responsibilities of their parents being met (Article 5), the right to not be separated from their parents against their will (Article 9); the right to be with their family (Article 10), and the right to have detention employed as a measure of last resort (Article 37) [33]. The evidence from this review indicates that violations of each of these articles result in deleterious physical and mental health outcomes. The review further supports the argument that a more theoretically solid foundation for the prohibition of the immigration detention of children is needed, based on article 37 of the UNRC – the right of the child to liberty [102]. Moreover, children who have experienced immigration detention should be considered a high-risk population with a need for intensive, dedicated and preventative interventions when their ultimate resettlement occurs [15].

### Meta-analysis findings: strengths and difficulties questionnaire (SDQ)

The total difficulties and sub-scale scores for the SDQ from the meta-analysis were approximately two- or more fold higher than comparable mainstream population means with the exception of the prosocial subscale.

#### SDQ total difficulties score – detained population vs. population norms

Our meta-analysis reveals a common mean score of 16.63 for the total Strengths and Difficulties Questionnaire (SDQ_total), categorising it as abnormal [74, 75, 103]. This figure is twice higher than that reported for mainstream populations in Australia, the United Kingdom, and the United States [104–106]. The normative Strengths and Difficulties Questionnaire (SDQ) data for children is contingent upon the method of collection, which may include parent, teacher, or self-reporting. Given that our meta-analysis relied on parent-reported SDQ data, reference will be made to these figures. Amongst 910 Australian children aged 7–17 across Victoria, the Mean (SD) Parent SDQ total difficulties was 8.2 (6.1). Amongst a representative British sample of 10,438 individuals aged between 5 and 15 [105], complete SDQ information was obtained from 10,298 parents (99% of the sample). Likewise, the normative mean (SD) for parent SDQ total difficulties was 8.4 (5.8). Lastly, normative SDQ data derived from the parents of 9878 American children aged 4–17 [106] noted a comparable mean (SD) SDQ total difficulties of 7.1 (5.7).

#### SDQ emotional symptoms score – detained population vs. population norms

Similarly, the meta-analytical emotional subscale (SDQ_emotion) score is classified as abnormal, exhibiting a common mean score of 5.40 [74, 75]. This is contrasted with a lower normative mean (SD) Parent SDQ emotional symptoms among mainstream Australian [2.1 (2.0)] [104], British [1.9 (2.0)] [105] and American children [1.6 (1.8)] [106].

#### SDQ conduct score – detained population vs. population norms

The meta-analytical conduct subscale (SDQ_Conduct) score exhibits a common mean score of 2.93 and is elevated compared to the mean/SD SDQ score of mainstream Australian 1.5(1.6) [104], British 1.6(1.7) [105], and American 1.3(1.6) children [106].

#### SDQ peer problems score – detained population vs. population norms

The meta-analytical peer problems subscale (SDQ_Peer) score exhibits a common mean score of 3.34 and is elevated compared to the mean/SD SDQ scores among mainstream Australian 1.6 (1.9) [104], British 1.5 (1.7) [105] and American 1.4 (1.5) [106] children.

#### SDQ Pro-social score – detained population vs. population norms

Conversely, the score for SDQ_pro_social is notably low at 6.60 when evaluated in comparison to mean/SD SDQ scores among mainstream Australian 8.3 (1.7) [104], British 8.6 (1.6) [105], and American 8.6 (1.8) [106] children. In the study conducted by MacLean et al., the sole subscale where the detained children exhibited superior mean scores compared to their community counterparts was the pro-social subscale, which includes behaviours such as empathy towards the feelings of others and participation in voluntary acts of assistance [19]. Increases in pro-social scores have been found in studies of children of parents with mental illness in the general community, and there is robust evidence for high rates of mental illness and psychological distress in adults in immigration detention [31, 32, 52, 53, 84, 89, 94, 107, 108]. The increase in pro-social scores of the detained children may, therefore, attest to the parentification and pseudo-maturity of children in a detention environment with high rates of mental disorders among parents and caregivers.

#### SDQ literature comparisons

The SDQ findings from this meta-analysis underscore broader research on the social and emotional well-being of refugee children. In contrast to our detained cohort, where SDQ total difficulties scores were consistently in the abnormal range, large population-based studies of resettled humanitarian children in Australia have found that 76%–94% function within the normal range of emotional and behavioural adjustment [109, 110]. The most common area of difficulty in non-detained refugee children was peer problems, particularly among girls and in caregiver-rated assessments. Yet, the rates remained substantially lower than those observed in detained children [110]. Further studies of non-detained refugee youth in the early post-resettlement period suggest that mental health difficulties may vary in intensity over time due to post-migration factors, such as parental psychopathology or older age [111]. This is consistent with our findings that detained children also face higher emotional symptoms scores, potentially exacerbated by parental distress and institutional trauma exposure.

In studies of children detained in US family immigration centres [112], similarly elevated SDQ scores were observed: over 75% reported emotional difficulties, with high rates of conduct, hyperactivity and peer problems. These scores correlated significantly with maternal trauma and mental illness, aligning with our meta-analytical interpretation that the mental health of detained children is closely entwined with family stress and trauma exposure. Lastly, longitudinal data indicate that protective factors — such as parental employment, stability (familial, financial, school, and housing), and external community support — may help improve SDQ outcomes over time for refugee children in the community [54]. This contrast reinforces the understanding that immigration detention undermines child wellbeing, not only through its direct impacts but also by disrupting access to the stabilising structures known to foster recovery and adjustment. Detained ASR children represent a particularly vulnerable group, with mental health profiles markedly worse than both non-detained refugee peers and population norms, and with recovery highly contingent on the presence of sustained protective supports.

### Overall implications

This systematic review collates the considerable pool of empirical evidence documenting the association between immigration detention and the risk of developing mental disorders in children, establishing it as an early-life adversity. Early life adversity is linked to numerous negative health outcomes, including the risk of neuropsychiatric illnesses [57]. Compared to adults, children may be at heightened risk for developing mental illnesses after exposure to trauma and adversity due to their brain’s developmental sensitivity [113]. This may predispose children to epigenetic, structural, and functional brain alterations [114, 115] that may ultimately lead to mental health sequelae persisting into adulthood [114].

Refugee children experience cumulative adversities throughout the processes of displacement, flight, and resettlement. Furthermore, immigration detention is linked to a multitude of additional adversities and violations of human rights [116]. It has been demonstrated that cumulative childhood trauma exposure predicts PTSD symptom complexity, specifically the tendency to exhibit symptoms of maladaptive attachment and greater dissociation [117].

### Strengths and limitations of the study

This systematic review and meta-analysis adhered to the methodologically rigorous PRISMA guidelines. A comprehensive search strategy, independent screening, data extraction, and quality appraisals were performed to ensure a systematic approach to synthesising the literature on this topic. This is the first systematic review and meta-analysis of its kind, to the best of the authors’ knowledge, examining the physical and mental health impacts of immigration detention on refugee and asylum-seeking children.

Relevant limitations primarily regarded the population studied. Firstly, the pooled detention sample was largely drawn from cross-sectional studies, preventing longitudinal analyses and restricting the differentiation between the impacts of detention and forced migration. Secondly, given the restricted nature of access to detained populations, data collection across the included studies was often opportunistic or drawn from convenience samples, meaning data needs to be interpreted with caution. Thirdly, only a small number of studies could be utilised for the meta-analysis, rendering sub-group analysis and meta-regression unsuitable given the small combined size. Gender-specific analyses were sparse in the primary literature, and UK/US studies overrepresented children in legal proceedings, potentially excluding less visible cohorts. Whilst Open Grey was included in the search, the review proper was restricted to peer-reviewed articles; information about trends in the detention of children and young people published in statistical reports published by governments or other organisations, indicative of international trends, may have been missed.

## Conclusion/implications

This systematic review and meta-analysis—the first to synthesise global evidence on the health impacts of immigration detention on asylum-seeking and refugee children and adolescents—reveals compelling evidence of detrimental physical, mental, neurodevelopmental, and psychosocial outcomes associated with detention. Key findings include acute and chronic health conditions (e.g., malnutrition, PTSD, developmental delays), with increasing severity observed with longer detention duration. However, the predominance of cross-sectional studies limits causal inferences and obscures definitive conclusions about long-term health trajectories. While sustained harm is plausible given the documented severity of impairments, longitudinal data are urgently needed to disentangle detention-specific effects from pre-migration trauma or post-release adversities. Despite these limitations, the consistency of harm across contexts underscores an ethical imperative: policymakers must prioritise alternatives to child and family detention, aligning with international human rights standards. Concurrently, healthcare systems should implement specialised, trauma-informed services for children post-detention to mitigate documented physical and mental health consequences. This evidence compels a dual mandate—prevention.

## Supplementary Information

Below is the link to the electronic supplementary material.

Supplementary file 1 (DOCX 2.02 MB)

## Data Availability

Data is provided within the manuscript or supplementary information files.

## Footnotes

1 Onshore detention in Australia refers to the detention of non-citizens within the country’s territory, including asylum seekers and individuals without valid visas. Offshore detention, on the other hand, involves the detention of asylum seekers in locations outside of Australia, namely Nauru and Papua New Guinea (Essex et al., 2023).
