# Supplementary material for: Immigration detention of children: a systematic review and meta-analysis of physical and mental health impacts
Source: Eur Child Adolesc Psychiatry. 2025 Aug 27;35(1):47–73. doi: 10.1007/s00787-025-02832-4 (PMC12916526; doi:10.1007/s00787-025-02832-4)

**Supplementary Files:**

**File 1 Quality Assessment of included studies**

**Analytical cross-sectional studies**

The review included ten analytical cross-sectional studies. Five defined sample inclusion criteria [6, 19, 60, 61, 77], while five lacked detail [32, 62, 82, 84, 85]. Nine studies detailed subjects and settings; one did not report demographic characteristics [62]. Nine studies used valid methods to assess exposure [6, 32, 60-62, 77, 82, 84, 88], while one study lacked a sufficient description of exposure assessment.

Criterion four of the JBI checklist (i.e., use of objective, standard criteria used for measurement of the condition) was not applicable to the studies included in this review. Three of the ten included studies identified and clearly stated confounding factors [84, 85, 88]. Of these, two described strategies to manage confounders [84, 85]. One study reported controlling for covariates but did not specify these covariates or the methods used for control [60]. Seven studies measured outcomes in a valid and reliable manner [19, 60-62, 77, 84, 85]. Two studies used less than adequate instruments to measure mental disorders [32, 82]. Specifically, Mares et al. (2016) [32] used the Kessler 10 (K-10) for depression and anxiety, a tool designed to screen for psychological distress; Derluyn et al. (2023) [82] assessed PTSD with an instrument that had not been previously validated. The remaining study attempted to establish the onset of conditions that had begun several years prior to the study’s commencement [6]. Six studies used appropriate statistical analyses [61, 62, 77, 82, 84, 85], while this criterion was inadequate for Ehntholt et al. (2018) [6]. The remaining three studies did not provide sufficient details regarding how statistical analysis was conducted [19, 60, 91].

**Prevalence studies**

Among the five prevalence studies included in this review all used restricted sample frames. Specifically, three studies employed a clinical population [43, 59, 83], while one limited their sample frame to individuals receiving specific legal assistance [81] and one to responding to a single charity for free legal aid [40]. Four studies [40, 59, 81] used an appropriate sampling method, while this criterion was unclear for Rothe (2002a) [83]. Based on the sample size calculation formula proposed by Daniel (1995) [124], none of the included studies used an appropriate sample size. All five studies provided detailed descriptions of subjects and settings. Data analysis was conducted with sufficient coverage of the identified sample in four studies [43, 59, 81, 83], while this criterion was unclear for Lorek al., (2009)[87]. All five studies employed valid methods to identify the condition/s of interest [40, 43, 59, 81, 83]. Three studies measured the condition/s in a standard, reliable way for all participants [43, 59, 81]. This criterion was unclear for Rothe (2002a), and Lorek et al. (2019) did not address this appropriately. Statistical analysis was inappropriate in two studies [40, 59] and insufficiently described in the remaining three [43, 81, 83]. All five studies had either an adequate response rate or managed the low response rate appropriately.

**Table 2: A quality review of 15 studies examining the mental and physical health of children in immigration detention [125]**

1. **ANALYTICAL CROSS-SECTIONAL STUDIES (n=10)**

| ***Study*** | **Inclusion Criteria** | **Subjects and Setting** | **Exposure** | **Condition** | **Identification of Confounders** | **Strategies for Confounders** | **Outcomes** | **Statistics** |
| --- | --- | --- | --- | --- | --- | --- | --- | --- |
| **Amarasena et al., 2022 [60]** | YES | YES | YES | N/A | UNCLEAR | UNCLEAR | YES | UNCLEAR |
| **Derluyn et al., 2023 [82]** | NO | YES | YES | N/A | NO | N/A | NO | YES |
| **Ehntholt et al., 2018 [6]** | YES | YES | YES | N/A | NO | N/A | NO | NO |
| **Essex et al., 2022 [62]** | NO | NO | YES | N/A | NO | N/A | YES | YES |
| **Maclean et al., 2019 [88]** | YES | YES | YES | N/A | YES | N/A | YES | UNCLEAR |
| **Mares et al., 2016 [32]** | NO | YES | YES | N/A | NO | N/A | NO | UNCLEAR |
| **Rothe et al., 2002 [85]** | NO | YES | UNCLEAR | N/A | YES | YES | YES | YES |
| **Sidamon-Eristoff et al., 2021 [84]** | NO | YES | YES | N/A | YES | YES | YES | YES |
| **Tosif et al., 2023 [61]** | YES | YES | YES | N/A | NO | N/A | YES | YES |
| **Zwi et al., 2017 [77]** | YES | YES | YES | N/A | NO | N/A | YES | YES |

1. **PREVALENCE STUDIES (n=5)**

| ***Study*** | **Sample frame** | **Sampling and Recruitment** | **Sample Size** | **Subjects and Setting Description** | **Data analysis conducted with sufficient Coverage** | **Valid methods used for identification of condition** | **Standard/Reliable Measure Of Condition** | **Statistical Analysis Appropriate** | **Adequate Response Rate or management of Low Response Rate** |
| --- | --- | --- | --- | --- | --- | --- | --- | --- | --- |
| **Hanes et al., 2019 [59]** | NO | YES | NO | YES | YES | YES | YES | NO | YES |
| **Lorek et al., 2009 [87]** | NO | YES | NO | YES | UNCLEAR | YES | NO | NO | YES |
| **Mares et al., 2004 [43]** | NO | YES | NO | YES | YES | YES | YES | UNCLEAR | YES |
| **Rothe et al., 2003 [83]** | NO | UNCLEAR | NO | YES | YES | YES | UNCLEAR | UNCLEAR | YES |
| **Steel et al., 2004 [81]** | NO | YES | NO | YES | YES | YES | YES | UNCLEAR | YES |

**File 2: Statistical Analysis**

**SDQ_emotion**

**SDQ_Conduct:**

**SDQ_Hyperactivity:**

**SDQ_Peer_problem:**

**SDQ_pro_social**

**SDQ_total:**

**Fig S2: Funnel plots for children mental health outcomes**

**SDQ_emotion**

**SDQ_Conduct:**

**SDQ_Hyperactivity:**

**SDQ_Peer_problem:**

**SDQ_pro_social**

**SDQ_total:**

**Fig S5: Leave-one-out analysis for children’s mental health outcomes**

**File 3: Search Terms**

Systematic Review Immigration Detention (Example of search)


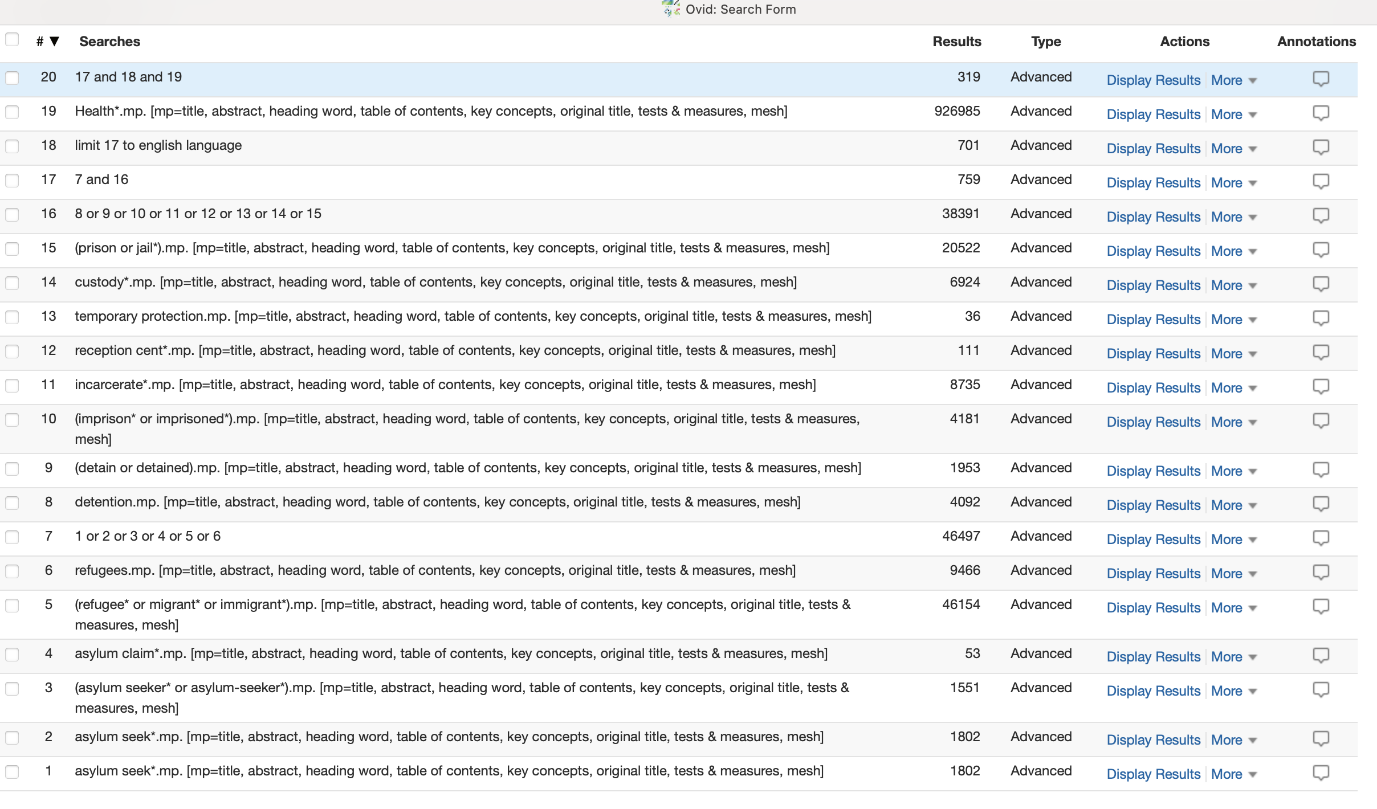


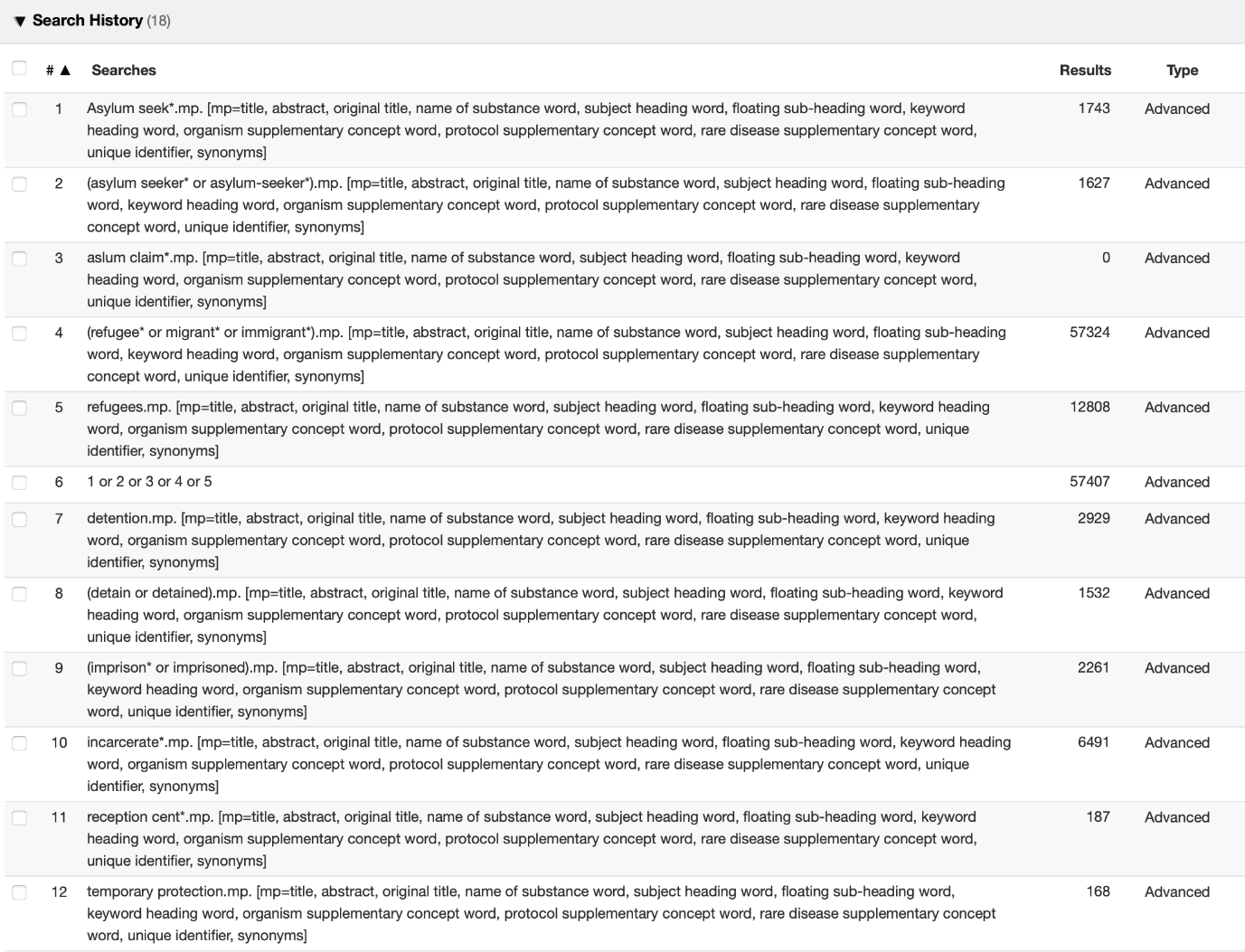


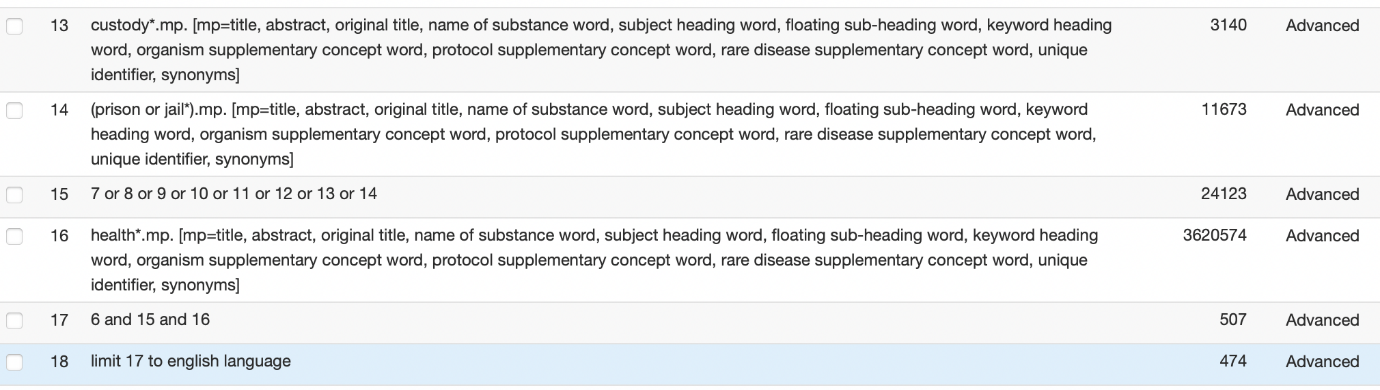


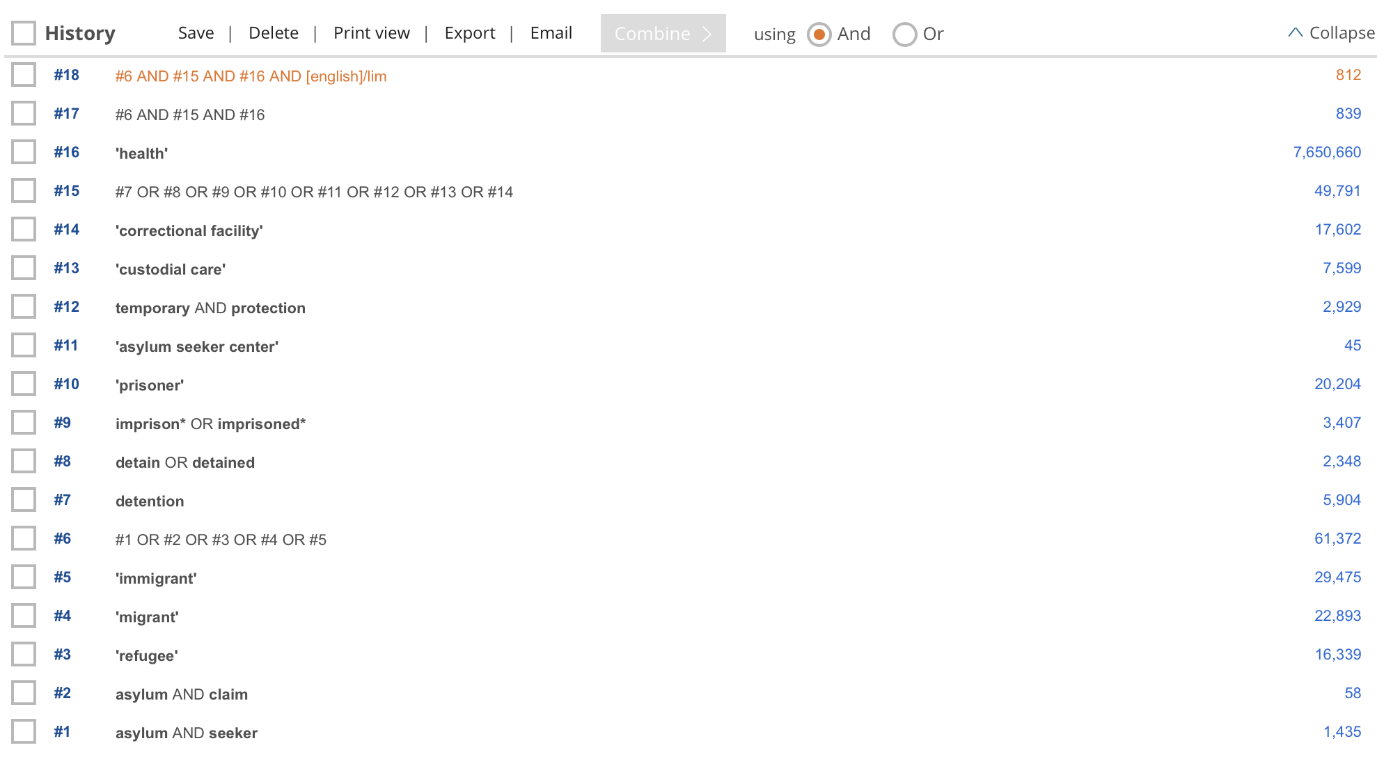

Supplement: Supplementary file 1 — (DOCX 2.02 MB) [file 787_2025_2832_MOESM1_ESM.docx]
